# Pharmacokinetics of Etravirine Combined with Atazanavir/Ritonavir and a Nucleoside Reverse Transcriptase Inhibitor in Antiretroviral Treatment-Experienced, HIV-1-Infected Patients

**DOI:** 10.1155/2015/938628

**Published:** 2015-01-15

**Authors:** Catherine Orrell, Franco Felizarta, André Nell, Thomas N. Kakuda, Ludo Lavreys, Steven Nijs, Lotke Tambuyzer, Rodica Van Solingen-Ristea, Frank L. Tomaka

**Affiliations:** ^1^Desmond Tutu HIV Foundation, UCT Medical School, P.O. Box 13801, Mowbray, Cape Town 7705, South Africa; ^2^Private Practice, 3535 San Dimas Street, Suite 24, Bakersfield, CA 93301, USA; ^3^Parexel International, University of the Free State, Campus Avenue South, Bloemfontein, Free State 9301, South Africa; ^4^Janssen Research & Development LLC, 1125 Trenton-Harbourton Road, Titusville, NJ 08560-0200, USA; ^5^Janssen Infectious Diseases BVBA, Turnhoutseweg 30, 2340 Beerse, Belgium

## Abstract

*Objectives*. TEACH (NCT00896051) was a randomized, open-label, two-arm Phase II trial to investigate the pharmacokinetic interaction between etravirine and atazanavir/ritonavir and safety and efficacy in treatment-experienced, HIV-1-infected patients. *Methods*. After a two-week lead-in of two nucleoside reverse transcriptase inhibitors (NRTIs) and atazanavir/ritonavir 300/100 mg, 44 patients received etravirine 200 mg bid with one NRTI, plus atazanavir/ritonavir 300/100 mg or 400/100 mg qd (*n* = 22 each group) over 48 weeks. *Results*. At steady-state etravirine with atazanavir/ritonavir 300/100 mg qd or 400/100 mg qd decreased atazanavir *C*_min⁡_ by 18% and 9%, respectively, with no change in AUC_24 h_ or *C*_max⁡_ versus atazanavir/ritonavir 300/100 mg qd alone (Day −1). Etravirine AUC_12 h_ was 24% higher and 16% lower with atazanavir/ritonavir 300/100 or 400/100 mg qd, respectively, versus historical controls. At Week 48, no significant differences were seen between the atazanavir/ritonavir groups in discontinuations due to adverse events (9.1% each group) and other safety parameters, the proportion of patients with viral load <50 copies/mL (intent-to-treat population, noncompleter = failure) (50.0%, atazanavir/ritonavir 300/100 mg qd versus 45.5%, 400/100 mg qd), and virologic failures (31.8% versus 27.3%, resp.). *Conclusions*. Etravirine 200 mg bid can be combined with atazanavir/ritonavir 300/100 mg qd and an NRTI in HIV-1-infected, treatment-experienced patients without dose adjustment.

## 1. Introduction

It is important to tailor the antiretroviral regimen for a treatment-experienced, HIV-1-infected patient so that it includes at least two (preferably three) fully active agents, based on pretreatment drug resistance testing. Other important considerations for combining antiretrovirals include side effects and drug-drug interactions [1–4].

Etravirine is a nonnucleoside reverse transcriptase inhibitor (NNRTI) for use in combination with other antiretrovirals in treatment-experienced patients with NNRTI-resistant HIV-1 virus. The HIV-1 protease inhibitor (PI) atazanavir is most commonly used with low-dose ritonavir in both treatment-naïve and -experienced patients [1–4].

In healthy volunteers who received etravirine 800 mg twice daily (bid) (formulation TF035) and atazanavir/ritonavir 300/100 mg once daily (qd), atazanavir area under the plasma concentration-time curve from time of administration to 24 hours after dosing (AUC_24 h_) decreased by 14% and the minimum plasma concentration (*C*_min⁡_) decreased by 38%. Etravirine AUC_24 h_ and *C*_min⁡_ both increased by 1.3-fold [5]. Although this Phase I study evaluated the 800 mg bid formulation, subsequent assessments demonstrated that exposure to etravirine was comparable between the commercial 100 mg tablet formulation (F060) when dosed 200 mg bid and the 200 mg formulation (TF035) when dosed 800 mg bid [6].

There is concern that a reduction in *C*_min⁡_ for boosted PIs can lower their antiviral activity [7, 8]. However, other studies have not demonstrated a clear relationship between atazanavir *C*_min⁡_ and virologic response [9–15], except for a recent study of unboosted atazanavir [16]. In the Phase III DUET trials in which etravirine was combined with darunavir/ritonavir and nucleoside/tide reverse transcriptase inhibitors (N[t]RTIs) [17–20], there was no apparent relationship between etravirine pharmacokinetics and virologic response or safety [21].

TEACH (trial with etravirine and atazanavir/ritonavir combination therapy for HIV infection; TMC125-TiDP2-C238; NCT00896051) was an exploratory trial to investigate whether the interaction between etravirine and atazanavir that has been observed in healthy volunteers also occurred in HIV-1-infected, treatment-experienced patients. The Week 2 pharmacokinetic results and final 48-week efficacy and safety analysis are presented.

## 2. Methods

### 2.1. Patients

The main inclusion criteria were HIV-1-infected adults who had previously received ≥1 antiretroviral regimen but failed ≤1 PI-based regimen and were currently on a stable but failing antiretroviral regimen (viral load [VL] > 500 copies/mL at screening) (Cobas Amplicor HIV-1 VL assay, Roche Molecular Systems, Inc., Branchburg, NJ, USA; assay performed by Covance Central Laboratory Services). Patients were required to be sensitive to atazanavir, etravirine, and the selected NRTIs (with the exception of emtricitabine or lamivudine) based on resistance testing at screening using the PhenoSense GT assay (Monogram Biosciences, South San Francisco, CA, USA). The assay combines phenotypic test results (PhenoSense HIV) with genotypic results (GeneSeq HIV) resulting in a net assessment interpretation on the report per tested drug, that is, resistant, partially sensitive, or sensitive. The clinical cut-offs used for etravirine and boosted atazanavir were 2.9–10.0 and 5.2, respectively. Exclusion criteria included HIV-2 infection, previous failure of ≥2 PI-containing regimens, and presence of ≥1 of the following NNRTI resistance-associated mutations (RAMs): K103R, V106I, I167V, V179D/F/T, Y181I/V, and G190S.

The trial protocol was reviewed and approved by an independent ethics committee and an institutional review board. The trial was carried out in accordance with the principles of Good Clinical Practice and Declaration of Helsinki. Written informed consent was obtained from all patients prior to study start.

### 2.2. Study Design and Treatment

TEACH was a randomized, open-label, two-arm, 48-week, Phase II trial to evaluate the pharmacokinetics, safety and tolerability, efficacy and virology of etravirine (F060), and atazanavir/ritonavir at two doses, both given with an NRTI over 48 weeks, in treatment-experienced, HIV-1-infected patients.

**Figure 1:**
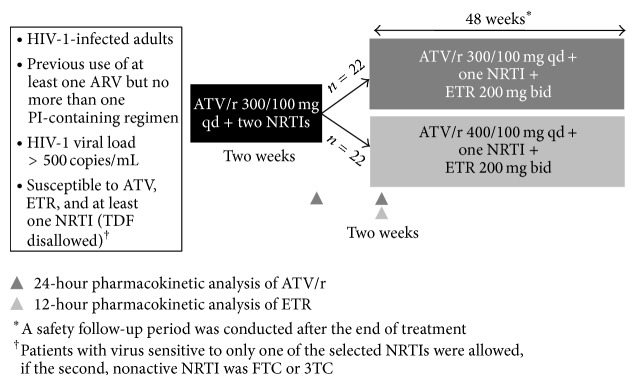
TEACH study design. TEACH was an open-label, randomized, exploratory Phase II trial. A two-week pretreatment period was followed by 48 weeks of treatment. 3TC: lamivudine; ARV: antiretroviral; ATV/r: atazanavir/ritonavir; ETR: etravirine; FTC: emtricitabine; NRTI: nucleoside reverse transcriptase inhibitor; PI: protease inhibitor; TDF: tenofovir disoproxil fumarate.

The trial consisted of a ≤4-week screening period, a pretreatment period of two weeks (atazanavir/ritonavir 300/100 mg qd and two NRTIs), and a 48-week treatment period. Patients were randomized 1 : 1, by a computer-generated interactive web response system, to receive either atazanavir/ritonavir 300/100 mg qd or 400/100 mg qd (Figure 1). Atazanavir/ritonavir was dosed at 300/100 mg qd to investigate if the interaction in treatment-experienced patients is consistent with that observed in healthy volunteers [5]. Atazanavir/ritonavir 400/100 mg qd was given to the other group to offset the potential reduction in atazanavir exposure, while at the same time investigating if the higher dose of atazanavir might further affect etravirine exposure. At baseline, in both groups, one of the two NRTIs was switched to etravirine 200 mg bid. NRTIs were selected by the investigators based on resistance testing; however, tenofovir disoproxil fumarate (TDF) was not permitted. Patients who were intolerant to their NRTI could switch to another sensitive NRTI, excluding TDF. Tenofovir decreases both atazanavir [22] and etravirine [23] exposure. These decreases do not significantly affect exposure of either drug, but an additive effect could result in reduced clinical efficacy when given in combination, particularly since etravirine also reduces atazanavir/ritonavir exposure. As such, a substudy to assess the addition of TDF for seven days on atazanavir and etravirine pharmacokinetics was conducted in patients with >24 weeks of treatment and who had ≥2 consecutive VL values < 50 copies/mL.

### 2.3. Study Objectives and Assessments

The primary objective of TEACH was to evaluate the pharmacokinetic interaction between etravirine and atazanavir/ritonavir at two different doses over 48 weeks. Secondary objectives included evaluation of safety and tolerability, antiviral activity, changes in CD4^+^ cell count, and viral genotype and phenotype.

Blood samples for analyses of atazanavir and ritonavir plasma concentrations were collected over 24 hours (before dose and 1, 2, 3, 4, 6, 9, 12, and 24 hours after dose) on the last day of the two-week pretreatment period and after Week 2 of the treatment period. Blood samples for evaluation of etravirine plasma concentrations were collected after Week 2. Atazanavir, ritonavir, and etravirine [24, 25] were assayed using validated liquid chromatography-mass spectrometry/mass spectrometry, with a lower limit of quantification of 250 ng/mL, 5.00 ng/mL, and 2.00 ng/mL, respectively.

Adverse events (AEs; division of AIDS grading [26]), laboratory parameters (serum clinical chemistry, hematology, and urinalysis), and 12-lead electrocardiograms (ECGs) were recorded throughout the study.

During the treatment session, plasma VL was determined at baseline, Weeks 2 and 4, and monthly until Week 48. Virologic failures were classified as patients who were still in the study at Week 12 and (i) who never achieved two consecutive VL values < 50 copies/mL (nonresponders) or (ii) first achieved two consecutive VL values < 50 copies/mL followed by two consecutive VL values > 50 copies/mL or discontinued for any reason with VL > 50 copies/mL (rebounders). Changes in viral phenotype and genotype in virologic failures were determined using the PhenoSense GT assay.

### 2.4. Data Analyses

Pharmacokinetic parameters were obtained using noncompartmental analysis (WinNonlin Professional version 4.1, Pharsight, Mountain View, CA, USA) and included *C*_min⁡_, maximum plasma concentration (*C*_max⁡_), and AUC_12 h_ for etravirine and AUC_24 h_ for atazanavir and ritonavir.

Statistical analysis was performed for atazanavir/ritonavir 300/100 mg qd or 400/100 mg qd with etravirine at Week 2 (test) versus atazanavir/ritonavir 300/100 mg qd without etravirine on the last day of the two-week pretreatment period (reference), including all observations for test and reference, paired and unpaired. The least square means (LSM) of *C*_min⁡_, *C*_max⁡_, and AUC_24_ for atazanavir and ritonavir were estimated with a linear mixed-effects model, including treatment as fixed effect and subject as a random effect (SAS version 9.3, SAS Institute, Cary, NC, USA). A 90% confidence interval (CI) was constructed around the difference between the LSM of test and reference. The LSM differences and 90% CIs were retransformed to the original scale. No effect (prespecified) was declared if the 90% CIs of the LSM ratio were within the limits of 80.00–125.00%. The pharmacokinetics of etravirine was compared with historic data from DUET [21].

Based on the atazanavir *C*_min⁡_ variability in HIV-infected patients receiving atazanavir/ritonavir 300/100 mg qd, 19 evaluable patients per dose group were required to provide sufficient precision for the estimated *C*_min⁡_ LSM ratio (90% CI within 20% of the estimate). Four additional patients per group were included to cover for early dropouts.

Safety analyses were conducted on the safety intent-to-treat (ITT) population, consisting of all patients who had taken at least one dose of trial medication (etravirine or atazanavir/ritonavir) (i.e., started the pretreatment session). QT intervals were corrected for heart rate according to Fridericia (QTcF).

Efficacy analyses were conducted on the efficacy ITT population, consisting of all patients who had taken at least one dose of etravirine during the trial (i.e., started the treatment session). The primary efficacy endpoint was virologic response at Week 48 (proportion of patients with VL < 50 copies/mL). For discontinuations, subsequent timepoints were imputed with baseline values (noncompleter status equals failure [NC = F]); intermediate missing values were imputed using last observation carried forward. Based on a logistic regression model with prebaseline plasma VL as covariate and treatment as factor, the predicted proportion of responders (with 95% CI) for each dose group was calculated. In addition, an ANCOVA model was used to analyze changes in plasma VL from prebaseline. Mean (standard error) change from prebaseline in CD4^+^ cell count (NC = F) was calculated.

## 3. Results

### 3.1. Patient Disposition and Baseline Characteristics

Seventeen investigators in four countries (Argentina, South Africa, Thailand and the USA) participated.

**Figure 2:**
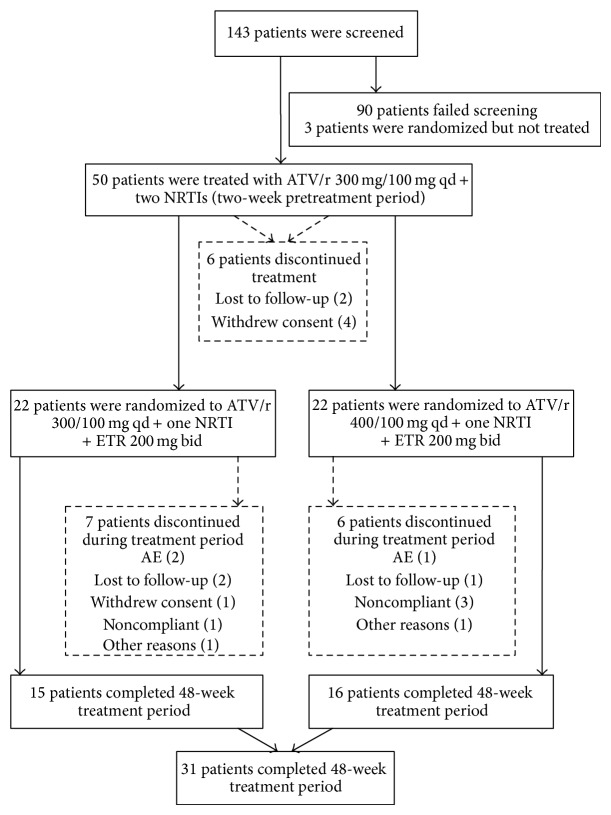
Patient disposition. AE: adverse event; ATV/r: atazanavir/ritonavir; ETR: etravirine; NRTI: nucleoside reverse transcriptase inhibitor.

Of the 143 screened patients, 50 were enrolled and treated with atazanavir/ritonavir 300/100 mg qd plus two NRTIs in the pretreatment session (Figure 2) (three patients were not treated). Of the 90 screening failures, the majority were due to VL ≤ 500 copies/mL at screening (*n* = 37), lack of sensitivity to etravirine, atazanavir and ≥1 NRTI (*n* = 19), and absence of ≥1 NNRTI RAM (*n* = 17). Six patients discontinued (Figure 2) and 44 patients continued into the 48-week treatment session (*n* = 22 in each atazanavir dose group). Thirty-one patients (62%) completed the study, with 13 patients discontinuing for noncompliance (*n* = 4), AEs (*n* = 3), loss to follow-up (*n* = 3), withdrawal of consent (*n* = 1), and other reasons (*n* = 2).

Baseline characteristics were generally balanced between treatment groups. There were, however, more Black/African-American patients and more patients from South Africa but fewer Asian patients in the atazanavir/ritonavir 400/100 mg qd group than in the atazanavir/ritonavir 300/100 mg qd group (Table 1). Of the patients with data available, most patients who continued into the treatment period had ≥2 NNRTI RAMs (81% [34/42]) [27] and ≥1 IAS-USA NRTI RAM (90.5% [38/42]) [28] but no IAS-USA primary PI mutations (88.1% [37/42]) at baseline [28].

### 3.2. Prior and Concomitant Antiretroviral Use

Most patients (90.9% [40/44]) entering the 48-week treatment session had previously received ≥1 NNRTI. All patients had previously received ≥2 NRTIs, and 34.1% of patients (15/44) had previously received a PI. During the 48-week treatment session, the most frequently used NRTIs were stavudine (36.4% [16/44]) and zidovudine (31.8% [14/44]). Use of NRTIs was similar between treatment groups.

### 3.3. Pharmacokinetics

**Figure 3:**
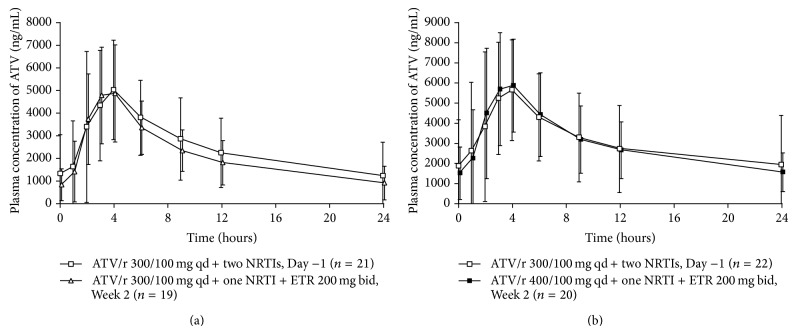
Mean ± standard deviation atazanavir plasma concentration-time curves after administration of atazanavir/ritonavir 300/100 mg qd for two weeks in combination with two NRTIs (Day −1), followed by administration of etravirine 200 mg bid for two weeks in combination with one NRTI and (a) atazanavir/ritonavir 300/100 mg qd or (b) atazanavir/ritonavir 400/100 mg qd (Week 2). ATV/r: atazanavir/ritonavir; bid: twice daily; ETR: etravirine; NRTI: nucleoside reverse transcriptase inhibitor; qd: once daily.

With atazanavir/ritonavir 300/100 mg qd, mean atazanavir plasma concentrations in the presence of etravirine (Week 2) were similar to the mean concentrations with atazanavir/ritonavir 300/100 mg qd alone (Day −1) over the first 4 hours but were slightly lower in the presence versus absence of etravirine from 4 to 24 hours (Figure 3(a)). Coadministration of etravirine with atazanavir/ritonavir 300/100 mg qd decreased atazanavir *C*_min⁡_ by 18% (Table 2) and *C*_max⁡_ and AUC_24 h_ were relatively unchanged (Table 2) compared with atazanavir/ritonavir 300/100 mg qd alone.

With atazanavir/ritonavir 400/100 mg qd, mean atazanavir plasma concentrations in the presence of etravirine (Week 2) were similar to mean concentrations with atazanavir/ritonavir 300/100 mg qd alone (Day −1) across the dosing interval (Figure 3(b)). Etravirine coadministration decreased atazanavir *C*_min⁡_ by 9%, and *C*_max⁡_ and AUC_24 h_ were relatively unchanged (Table 2) versus atazanavir/ritonavir 300/100 mg qd alone.

**Figure 4:**
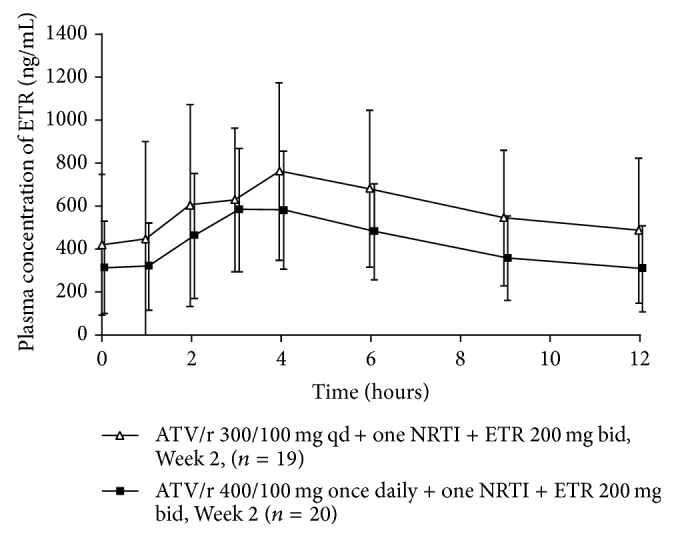
Mean ± standard deviation etravirine plasma concentration-time curves after administration of etravirine 200 mg bid for two weeks in combination with atazanavir/ritonavir 300/100 mg qd or 400/100 mg qd and one NRTI. ATV/r: atazanavir/ritonavir; bid: twice daily; ETR: etravirine; NRTI: nucleoside reverse transcriptase inhibitor; qd: once daily.

Mean etravirine plasma concentrations were higher in the atazanavir/ritonavir 300/100 mg qd group than in the atazanavir/ritonavir 400/100 mg qd group, across the dosing interval (Figure 4). Compared with historic control data [21], coadministration of atazanavir/ritonavir 300/100 mg qd with etravirine resulted in 7%, 6%, and 24% increases in etravirine *C*_min⁡_, *C*_max⁡_, and AUC_12 h_, respectively (Table 3). With atazanavir/ritonavir 400/100 mg qd, there were 17%, 13%, and 16% decreases in etravirine *C*_min⁡_, *C*_max⁡_, and AUC_12 h_, respectively, compared with historic data (Table 3).

Seven patients participated in the pharmacokinetic substudy, 3 patients randomized to atazanavir/ritonavir 300/100 mg qd and 4 patients randomized to atazanavir/ritonavir 400/100 mg qd. Because of the small sample size, results of the two groups were combined. Adding TDF to atazanavir/ritonavir and etravirine decreased atazanavir *C*_min⁡_ by 25% (LSM ratio [90% CIs]: 0.75 [0.41–1.36]), *C*_max⁡_ by 3% (0.97 [0.75–1.25]), and AUC_24 h_ by 9% (0.91 [0.72–1.13]) and decreased etravirine *C*_max⁡_ by 19% (0.81 [0.72–0.91]) and AUC_12 h_ by 15% (0.85 [0.79–0.92]), compared with Day −1. Etravirine *C*_min⁡_ was relatively unchanged in the presence of TDF (LSM ratio [90% CI]: 0.97 [0.85–1.10]).

### 3.4. Safety and Tolerability

During the 48-week treatment session, safety and tolerability were comparable between the two treatment groups (Table 4). The most common AEs were cough, headache, influenza, and sinusitis (Table 4). Each of the AEs considered at least possibly related to etravirine (not including laboratory abnormalities reported as an AE), that is, pruritus, rash, dyspepsia, nausea, hyperuricemia, goitre, edema, and headache, occurred in no more than one patient overall.

The majority of AEs were Grade 1 or 2 in severity. Each Grade 3 or 4 AE or serious AE was reported in only one patient, except for pneumonia, which occurred in one patient in each treatment group. Grade 4 AEs were reported in three patients in the atazanavir/ritonavir 300/100 mg qd group, only one of which (hyperuricemia) was considered possibly related to etravirine.

No serious AEs were considered related to etravirine. One death occurred in the atazanavir/ritonavir 300/100 mg qd group (Table 4) as a result of metastatic malignant melanoma (considered not related to etravirine). Except for an increase in direct, indirect, and total bilirubin values during the pretreatment period, which remained elevated throughout the Week 48 treatment period, mean changes over time in clinical laboratory parameters were generally small in both treatment groups and not considered clinically relevant. No clinically relevant mean changes from baseline were seen in vital signs or ECG parameters. No patients had a QTcF interval ≥ 480 ms.

### 3.5. Antiviral Activity and Development of Resistance

**Figure 5:**
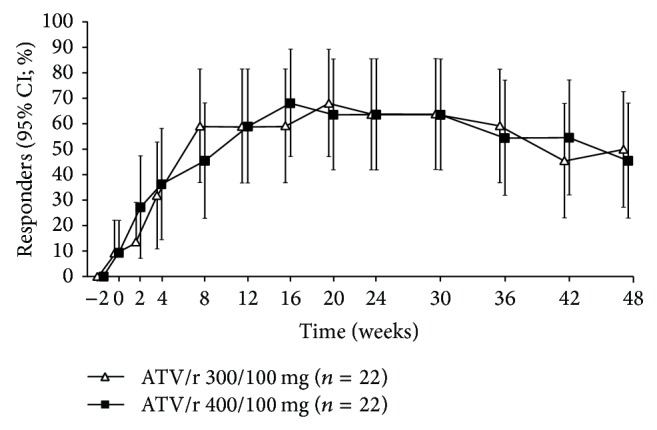
Virologic response (viral load (VL) < 50 copies/mL) over time to Week 48 (intent-to-treat noncompleter equals failure: NC = F). NC = F missing values after discontinuation imputed with change = 0. Last observation was carried forward otherwise. ATV/r: atazanavir/ritonavir; CI: confidence interval; VL: viral load.

Virologic response (VL < 50 copies/mL; ITT, NC = F) was achieved in 50% (95% CI: 28.2–71.8%) of patients in the atazanavir/ritonavir 300/100 mg qd group and 45.5% (95% CI: 24.4–67.8%) in the atazanavir/ritonavir 400/100 mg qd group (Table 5). At Week 48, there was no difference in virologic response between the two treatment groups when adjusted for prebaseline VL (*P* = 0.692; logistic regression) or in the mean change in log_10_ plasma VL from prebaseline (*P* = 0.845; ANCOVA). The snapshot analysis results and the NC = F analysis results were similar (Table 5). In the snapshot analysis, five patients (11.4%) had discontinued the study by Week 48 for reasons not related to safety or efficacy and with the last available VL < 50 copies/mL. In both treatment groups, the virologic response (NC = F) increased from prebaseline (Week −2) to Week 20, remained stable until Week 30, and decreased slowly from Week 30 to 48 (Figure 5).

Week 48 virologic failure rates were comparable between treatment groups (31.8% [7/22] in the atazanavir/ritonavir 300/100 mg qd group and 27.3% [6/22] in the atazanavir/ritonavir 400/100 mg qd group (four nonresponders and nine rebounders)).

In the 13 virologic failures, nine NNRTI RAMs emerged at endpoint (V90I, K101P, E138G, Y181C, V189I, G190S, H221Y, P225H, and M230L). Seven of these RAMs occurred in one patient each, whereas K101P developed in two patients and Y181C emerged in three patients. Median etravirine weighted genotypic score (*n* = 13) increased from 0 (range: 0–3) at baseline to 2.5 (0–4.5) at endpoint and median etravirine FC (*n* = 12) increased from 0.73 (0.4–1.7) to 2.99 (0.6–40.0), whereas no increase in atazanavir FC was observed, and the NRTIs used remained sensitive. Emerging atazanavir RAMs [27] were only observed in the atazanavir/ritonavir 300/100 mg qd group: L10F (*n* = 1), V32I (*n* = 1), M46I (*n* = 2), and V82A (*n* = 1). No primary PI mutations associated with resistance to atazanavir emerged at endpoint.

### 3.6. Immunologic Response

An increase in CD4^+^ cell count from prebaseline was observed at all timepoints in both treatment groups. There was no significant difference (*P* = 0.714; ANCOVA) in the mean increase from prebaseline in absolute CD4^+^ count (NC = F) at Week 48 between treatment groups (Table 5).

## 4. Discussion

In HIV-1-infected patients who have failed virologically with demonstrated RAMs on an antiretroviral regimen, treatment options for the new regimen include a fully active PI/r and classes not used previously, for example, a fusion or integrase inhibitor, CCR5 antagonist (R5 virus only), or an NNRTI such as etravirine, based on genotypic drug resistance testing. As such, the TEACH study sought to investigate the interaction between etravirine and atazanavir/ritonavir at two different doses in treatment-experienced, HIV-1-infected patients. We showed that etravirine 200 mg bid can be combined with atazanavir/ritonavir 300/100 mg qd and an NRTI in a treatment regimen for HIV-1-infected, treatment-experienced patients without dose adjustment.

An earlier drug-drug interaction study showed a marked decrease in atazanavir exposure when given with etravirine [5]. The current study, in treatment-experienced, HIV-1-infected patients, appeared to result in a smaller effect on atazanavir than observed in healthy volunteers [5]. The cause of this is unknown but may be related to differences in metabolic activity between healthy volunteers and HIV-infected patients. For example, CYP3A activity in HIV-infected patients is half of that observed in healthy volunteers; P-glycoprotein activity is also lower [29]. It could also be due to differences in the etravirine formulation (TF035 versus F060) as the commercial formulation F060 is more bioavailable [6]. An interaction study with etravirine administered as either of the two formulations and TDF, however, showed no significant difference between the two formulations [23]. Increasing the atazanavir/ritonavir dose from 300/100 to 400/100 mg qd resulted in a less than proportional increase in atazanavir exposure. However, a comparison between atazanavir/ritonavir dose groups of the changes in atazanavir pharmacokinetics in the presence of etravirine should be interpreted with caution as atazanavir exhibits nonlinear disposition [30].

The effect of atazanavir/ritonavir 300/100 mg qd on etravirine exposure in HIV-infected patients was also less than anticipated based on previous results in healthy volunteers [5]. Increasing the dose of atazanavir/ritonavir to 400/100 mg qd further reduced etravirine exposure. This observation should be interpreted with caution because the 95% CIs of the LSM ratios were wide and the effect of increasing the dose of atazanavir/ritonavir to 400/100 mg qd is based on a comparison between groups.

There were no clinically significant differences in safety between the two treatment groups. Following coadministration of etravirine and atazanavir/ritonavir, the majority of AEs were Grade 1 or 2. No new safety findings were observed compared with the known safety profiles of etravirine and atazanavir.

The virologic response rate was not significantly different between the atazanavir/ritonavir 300/100 mg qd and 400/100 mg qd groups. While the approved atazanavir/ritonavir dose in treatment-experienced adults is 300/100 mg qd [1–4], similar responses have been observed with atazanavir/ritonavir 300/100 mg qd and atazanavir 400 mg qd in treatment-naïve patients [31, 32]. In the current study, patients were highly treatment experienced, with 81% having ≥2 NNRTI RAMs at baseline. Virologic response was lower in this study than in Phase III DUET trials at Week 48, where a response of 57% was observed in treatment-experienced adults receiving etravirine combined with darunavir/ritonavir and N[t]RTIs and reusing or not using enfuvirtide [17–20]. However, virologic response in the current study was influenced by a relatively high proportion of discontinuations of patients with undetectable VL (in relation to a small total sample size) for reasons not related to safety or efficacy. Nonetheless, the observed response rates are in line with other studies in similar populations of highly treatment-experienced patients [33–35].

The only NNRTI RAMs emerging at endpoint in ≥1 patient experiencing virologic failure were K101P and Y181C, which were both previously defined as etravirine RAMs [36]. No relevant development of resistance to atazanavir occurred.

The main limitation of the study is that it was an exploratory Phase II study. Since the study was powered to evaluate the pharmacokinetic interaction between etravirine and atazanavir/ritonavir rather than to compare virologic outcomes between the two different atazanavir doses, the safety and efficacy findings should be interpreted with caution, noting the small numbers of patients in each treatment group. A second limitation is the large number of patients who discontinued the study, influencing the results. However, these discontinuations were mainly attributed to reasons other than AEs such as nonadherence, loss to follow-up, and withdrawal of consent.

A third limitation was that TDF was not permitted; therefore, the effect on efficacy due to its interactions with atazanavir [22] and etravirine [23], if TDF was to be coadministered with these agents, is not known. In the substudy, while addition of TDF to atazanavir/ritonavir and etravirine resulted in a modest decrease in the exposure of atazanavir and etravirine, this result should be interpreted with caution given the small number of patients.

In summary, there was a smaller pharmacokinetic interaction between etravirine and atazanavir/ritonavir in HIV-infected patients than what was observed previously in healthy volunteers [5]. Increasing the atazanavir dose to 400 mg did not substantially increase atazanavir exposure but decreased etravirine exposure. Etravirine 200 mg bid can be combined with atazanavir/ritonavir 300/100 mg qd and an NRTI, in a treatment regimen for HIV-1-infected, treatment-experienced patients without dose adjustment.

## Figures and Tables

**Table 1:** Patient baseline demographics and disease characteristics of patients who started the pretreatment session.

	Atazanavir/ritonavir 300/100 mg qd + one NRTI + etravirine 200 mg bid (*n* = 25)	Atazanavir/ritonavir 400/100 mg qd + one NRTI + etravirine 200 mg bid (*n* = 25)	All patients (*n* = 50)
Female [*n* (%)]	12 (48.0)	13 (52.0)	25 (50.0)
Age, years [median (range)]	39.0 (26–63)	40.0 (18–56)	40.0 (18–63)
Race [*n* (%)]			
Black or African-American	12 (48.0)	18 (72.0)	30 (60.0)
Asian	6 (24.0)	2 (8.0)	8 (16.0)
White	6 (24.0)	5 (20.0)	11 (22.0)
Others	1 (4.0)	0	1 (2.0)
Country [*n* (%)]			
South Africa	9 (36.0)	13 (52.0)	22 (44.0)
USA	10 (40.0)	8 (32.0)	18 (36.0)
Thailand	6 (24.0)	2 (8.0)	8 (16.0)
Argentina	0	2 (8.0)	2 (4.0)
Prebaseline log_10_⁡VL (copies/mL) [median (range)]	4.1 (2–6)	4.1 (2–6)	4.1 (2–6)
Prebaseline CD4^+^ cell count, median (range), cells/mm^3^	186 (8–678)	238 (55–1061)	223 (8–1061)
Duration of HIV infection, years (median (range))	7.2 (2–16)	6.7 (1–26)	7.0 (1–26)
CDC clinical stage of infection [*n* (%)]			
A	10 (40.0)	9 (36.0)	19 (38.0)
B	7 (28.0)	7 (28.0)	14 (28.0)
C	8 (32.0)	9 (36.0)	17 (34.0)
Mode of HIV infection [*n* (%)]			
Heterosexual contact	15 (60.0)	12 (48.0)	27 (54.0)
Heterosexual contact/MSM	1 (4.0)	1 (4.0)	2 (4.0)
MSM	5 (20.0)	5 (20.0)	10 (20.0)
Others	4 (16.0)	7 (28.0)	11 (22.0)

CDC: Centers for Disease Control and Prevention; MSM: men who have sex with men; *n*: number of patients per treatment group; *n* (%): number (proportion) of patients with specified parameter; NRTI: nucleoside reverse transcriptase inhibitor; VL: viral load.

Prebaseline: Day 1 of the pretreatment period.

**Table 2:** Pharmacokinetic parameters and statistical analysis of atazanavir after administration of atazanavir/ritonavir 300/100 mg qd for two weeks in combination with two NRTIs (Day −1), followed by administration of etravirine 200 mg bid for two weeks in combination with one NRTI and atazanavir/ritonavir 300/100 mg qd or atazanavir/ritonavir 400/100 mg qd (Week 2).

Pharmacokinetics of atazanavir mean ± SD; *t*_max⁡_: median (range)	Group one		Group two	
	ATV/r 300/100 mg qd + two NRTIs (reference) (*n* = 20^*^)	ATV/r 300/100 mg qd + one NRTI + ETR 200 mg bid (Test 1) (*n* = 18^†^)	ATV/r 300/100 mg qd + two NRTIs (reference) (*n* = 21^‡^)	ATV/r 400/100 mg qd + one NRTI + ETR 200 mg bid (Test 2) (*n* = 20^§^)
*C*_0 h_, ng/mL	1339 ± 1728	845.7 ± 703.3	1898 ± 2298	1545 ± 1296
*C*_min⁡_, ng/mL	1104 ± 1511	758.6 ± 610.5	1671 ± 2310	1107 ± 866.8
*C*_max⁡_, ng/mL	5652 ± 2735	5232 ± 2166	6419 ± 2853	6950 ± 2693
*t*_max⁡_, h	4.00 (1.98–6.00)	3.00 (1.00–4.17)	3.04 (1.00–6.00)	3.21 (1.25–6.17)
AUC_0–24 h_, ng·h/mL	60030 ± 39690	55070 ± 21860	74210 ± 55480	72220 ± 34600

LSM ratio (90% CI) of test versus reference^¶^				

*C*_min⁡_	0.82 (0.55–1.22)		0.91 (0.63–1.33)	
*C*_max⁡_	0.96 (0.80–1.16)		1.05 (0.86–1.27)	
AUC_0–24 h_	0.96 (0.76–1.22)		0.99 (0.81–1.21)	

ATV/r: atazanavir/ritonavir; AUC_0–24 h_: area under the plasma concentration-time curve from time 0 to 24 hours; bid: twice daily; CI: confidence interval; *C*_max⁡_: maximum plasma concentration; *C*_min⁡_: minimum plasma concentration; *C*_0 h_: predose plasma concentration; ETR: etravirine; LSM: least square mean; NRTI: nucleoside reverse transcriptase inhibitor; qd: once daily; SD: standard deviation; *t*_max⁡_: time to maximum plasma concentration.

^*^ *n* = 21 for *C*_0 h_, *n* = 19 for AUC_24 h_; ^†^ *n* = 19 for *C*_0 h_, *C*_max⁡_, and *T*_max⁡_; ^‡^ *n* = 22 for *C*_0 h_, *C*_max⁡_, and *t*_max⁡_; ^§^ *n* = 18 for *C*_min⁡_, *n* = 19 for AUC_24 h_;  ^¶^calculated based on log-transformed pharmacokinetic parameters.

**Table 3:** Pharmacokinetic parameters and statistical analysis of etravirine after administration of etravirine 200 mg bid for two weeks in combination with atazanavir/ritonavir 300/100 mg qd or 400/100 mg qd and one NRTI and from the pooled DUET pharmacokinetic substudies at Week 4 [21].

Pharmacokinetics of ETR mean ± SD; *t*_max⁡_: median (range)	Pooled DUET [21]: ETR 200 mg bid + DRV/r 600/100 mg bid, Week 4 (reference) (*n* = 25)	ATV/r 300/100 mg qd + one NRTI + ETR 200 mg bid, Week 2 (Test 1) (*n* = 18^*^)	ATV/r 400/100 mg qd + one NRTI + ETR 200 mg bid, Week 2 (Test 2) (*n* = 18^†^)
*C*_0 h_, ng/mL	545.0 ± 818.6	422.2 ± 327.9	316.6 ± 215.4
*C*_min⁡_, ng/mL	452.6 ± 806.0	425.1 ± 328.1	286.5 ± 198.0
*C*_max⁡_, ng/mL	879.6 ± 1030	773.0 ± 360.5	628.7 ± 294.0
*t*_max⁡_, h	3.95 (0.08–6.08)	4.00 (3.00–9.00)	4.00 (2.00–6.07)
AUC_0–12 h_, ng·h/mL	7964 ± 11180	7629 ± 4213	5171 ± 2695

LSM ratio (90% CI) of test versus reference^‡^			

		Test 1 versus reference	Test 2 versus reference

*C*_min⁡_	—	1.07 (0.60–1.90)	0.83 (0.54–1.29)
*C*_max⁡_	—	1.06 (0.78–1.46)	0.87 (0.65–1.18)
AUC_0–12 h_	—	1.24 (0.88–1.76)	0.84 (0.60–1.18)

ATV/r: atazanavir/ritonavir; AUC_0–12 h_: area under the plasma concentration-time curve from time 0 to 12 hours; bid: twice daily; CI: confidence interval; *C*_max⁡_: maximum plasma concentration; *C*_min⁡_: minimum plasma concentration; *C*_0 h_: predose plasma concentration; ETR: etravirine; LSM: least square mean; NRTI: nucleoside reverse transcriptase inhibitor; qd: once daily; SD: standard deviation; *t*_max⁡_: time to maximum plasma concentration.

^*^ *n* = 19 for *C*_0 h_, *n* = 16 for *C*_min⁡_, *n* = 17 for AUC_0–12 h_, ^†^ *n* = 19 for *C*_0 h_, and *n* = 20 for *C*_max⁡_ and *t*_max⁡_; ^‡^calculated based on log-transformed pharmacokinetic parameters.

**Table 4:** Overview of AEs and laboratory abnormalities during the 48-week treatment session.

Incidence, (*n* (%))	Atazanavir/ritonavir 300/100 mg qd + one NRTI + etravirine 200 mg bid (*n* = 22)	Atazanavir/ritonavir 400/100 mg qd + one NRTI + etravirine 200 mg bid (*n* = 22)	All patients (*n* = 44)
Any AE	20 (90.9)	17 (77.3)	37 (84.1)
Any treatment-related AE	5 (22.7)	5 (22.7)	10 (22.7)
Grade 3-4 AEs	4 (18.2)	4 (18.2)	8 (18.2)
AEs leading to discontinuation	2^*^ (9.1)	2^†^ (9.1)	4 (9.1)
Serious AEs	4 (18.2)	2 (9.1)	6 (13.6)
Death	1 (4.5)	0	1 (2.3)

AEs regardless of relationship to study treatment and occurring in ≥3 patients^‡^			
Cough	5 (22.7)	4 (18.2)	9 (20.5)
Headache	3 (13.6)	4 (18.2)	7 (15.9)
Influenza	2 (9.1)	3 (13.6)	5 (11.4)
Sinusitis	3 (13.6)	0	3 (6.8)

^§^AEs of interest regardless of relationship to study treatment, occurring in ≥2 patients			
Hepatotoxicity	5 (22.7)	5 (22.7)	5 (22.7)
Rash (any type)^¶^	3 (13.6)	2 (9.1)	5 (11.4)
Neuropsychiatric AEs	3 (13.6)	0	3 (6.8)

Treatment-emergent Grade 2–4 laboratory abnormalities occurring in ≥2 patients			
	*n* = 21	*n* = 21	*n* = 42^¥^

Hyperbilirubinemia	8 (38.1)	6 (28.6)	14 (33.3)
Increased total cholesterol	3 (14.3)	3 (14.3)	6 (14.3)
Increased LDL-cholesterol	1 (4.8)	5 (23.8)	6 (14.3)
Hyperglycemia	4 (19.0)	1 (4.8)	5 (11.9)
Increased pancreatic amylase	1 (4.8)	3 (14.3)	4 (9.5)
Increased segmented neutrophils	2 (9.5)	2 (9.5)	4 (9.5)
Increased hemoglobin	1 (4.8)	1 (4.8)	2 (4.8)
Increased white blood cell count	2 (9.5)	0	2 (4.8)
Increased AST	1 (4.8)	1 (4.8)	2 (4.8)

AE: adverse event; AST: aspartate amino transferase; LDL: low-density lipoprotein; *n*: number of patients per treatment group; *n* (%): number (proportion) of patients with specified parameter; NRTI: nucleoside reverse transcriptase inhibitor.

^*^One patient died (metastatic malignant melanoma), and one experienced Grade 2 maculopapular rash probably related to etravirine and atazanavir and discontinued treatment on the day of onset (eight days after the start of etravirine treatment).

^†^One patient discontinued at Week 48 due to Grade 2 secondary syphilis considered not related to study medication. Another patient became pregnant and discontinued the study.

^‡^Not including laboratory abnormalities reported as an AE.

^§^AEs of special interest were selected based on their association with other antiretrovirals and relevance based on preclinical or earlier clinical data and the target population. Hepatotoxicity was considered at least possibly related to atazanavir for five and four patients in the atazanavir/ritonavir 300/100 mg qd and 400/100 mg qd treatment groups, respectively and at least possibly related to etravirine in three patients. Neuropsychiatric AEs of interest, including Grade 2 depression in one patient and Grade 2 peripheral neuropathy in two patients, were considered not serious and not related to etravirine and did not lead to discontinuation.

^¶^Grouped term including rash (not further specified), maculopapular rash and papular rash.

^*¥*^Two patients did not have post-dose laboratory data because they discontinued shortly after baseline.

**Table 5:** Virologic and immunologic outcomes at Week 48.

Week 48 outcomes	Atazanavir/ritonavir 300/100 mg qd + one NRTI + etravirine 200 mg bid (*n* = 22)	Atazanavir/ritonavir 400/100 mg qd + one NRTI + etravirine 200 mg bid (*n* = 22)	All patients (*n* = 44)
ITT, NC = F			
^*^VL < 50 copies/mL, *n* (%)	11 (50.0)	10 (45.5)	21 (47.7)
VL < 400 copies/mL, *n* (%)	11 (50.0)	13 (59.1)	24 (54.5)
^†^log_10_⁡VL, mean (SE) change from prebaseline (Week −2)	−1.4 (0.24)	−1.4 (0.29)	−1.4 (0.18)
^‡^Increase from prebaseline in absolute CD4^+^ count, cells/mm^3^ (mean (SE)) (NC = F)	105 (31.1)	132 (32.6)	118 (22.3)
Snapshot			
VL < 50 copies/mL [*n* (%)]	11 (50.0)	10 (45.5)	21 (47.7)

^*^ *P* = 0.692, logistic regression; ^†^ *P* = 0.845, ANCOVA; and ^‡^ *P* = 0.714, ANCOVA for comparison between the two treatment groups, corrected for baseline VL and baseline CD4^+^ count respectively.

ITT: intent-to-treat; *n*: number of patients per treatment group; *n* (%): number (proportion) of patients with observations; NC = F: noncompleter equals failure (missing values after discontinuation imputed with change = 0; last observation was carried forward otherwise); NRTI: nucleoside reverse transcriptase inhibitor; SE: standard error; VL: viral load.
